# Extremophilic SHMTs: From Structure to Biotechnology

**DOI:** 10.1155/2013/851428

**Published:** 2013-06-13

**Authors:** Sebastiana Angelaccio

**Affiliations:** Dipartimento di Scienze Biochimiche “A. Rossi Fanelli,” “Sapienza” Università di Roma, Piazzale Aldo Moro 5, 00185 Roma, Italy

## Abstract

Recent advances in molecular and structural biology have improved the availability of virtually any biocatalyst in large quantity and have also provided an insight into the detailed structure-function relationships of many of them. These results allowed the rational exploitation of biocatalysts for use in organic synthesis. In this context, extremophilic enzymes are extensively studied for their potential interest for many biotechnological and industrial applications, as they offer increased rates of reactions, higher substrate solubility, and/or longer enzyme half-lives at the conditions of industrial processes. Serine hydroxymethyltransferase (SHMT), for its ubiquitous nature, represents a suitable model for analyzing enzyme adaptation to extreme environments. In fact, many SHMT sequences from Eukarya, Eubacteria and Archaea are available in data banks as well as several crystal structures. In addition, SHMT is structurally conserved because of its critical metabolic role; consequently, very few structural changes have occurred during evolution. Our research group analyzed the molecular basis of SHMT adaptation to high and low temperatures, using experimental and comparative *in silico* approaches. These structural and functional studies of SHMTs purified from extremophilic organisms can help to understand the peculiarities of the enzyme activity at extreme temperatures, indicating possible strategies for rational enzyme engineering.

## 1. Introduction 

 Studies on protein stability represented an important issue in the past forty years, owing to the central role these macromolecules play in maintaining life and their involvement in many diseases affecting humans. The comparison of structural and functional features of proteins among thermophilic/psychrophilic organisms and their homologs from mesophilic counterparts can provide insights into the ability of extremophiles to function at their extreme habitat temperatures and may give clues to better define the forces that stabilize proteins. In case of adaptations to extremes of pH, salinity, and pressure, membrane components and protective small molecules often play an important role and have been studied quite extensively [1–3]. For temperature adaptation, however, environmental stress generally cannot be avoided by compensatory mechanisms, and thus the cellular components themselves, specifically the proteins, have to achieve a certain level of stability at extreme temperatures, at which most of living species cannot grow because of their inability to maintain adequate metabolic fluxes. For this reason, much interest has been directed to understand how proteins from thermophilic/psychrophilic organisms retain their structure and function at high or low temperatures, respectively. In particular, enzymes perform important tasks in all biological systems, and they do so by maintaining a specific globular conformation. This functional state, called the native state, is stabilized in a balancing act of opposing forces. The players in this act have long been identified [4], although their relative contributions have been debated [5–9]. The major stabilizing forces include the hydrophobic effect and hydrogen bonding, while conformational entropy favors the unfolded state. The crystal structures of extremophilic enzymes unambiguously indicate a continuum in the molecular adaptations to temperature. For example, from psychrophiles (living at low temperatures close to 0°C) to mesophiles (living at intermediate temperatures close to 37°C) and to thermophiles (living at high temperatures above to 37°C), there is a clear increase in the number and strength of all known weak interactions and structural factors, such as hydrophobicity, polar surface area of the molecules, involved in protein stability [10–13]. Therefore, the same mechanism of molecular adaptation is involved in response to two distinct selective pressures, that is, the requirement for stable protein structure and activity in thermophiles and the requirement for high enzyme activity in psychrophiles. This of course suggests intricate and still controversial relationships between activity and stability in these naturally evolved enzymes. It seems that each extremophilic enzyme adopts its own adaptive strategy. In this contest, SHMT, for its ubiquitous nature and its critical metabolic role, represents a paradigm to study enzymes' adaptations to extreme environments.

 The discovery of new extremophilic microorganisms and their enzymes had a great impact on the field of biocatalysis. The industrial application of enzymes that can withstand harsh conditions has greatly increased over the past decade. Recent advances in the study of extremozymes point to the acceleration of this trend. Much of the biotechnological interest in enzymes from extremophilic organisms stems from their surprising properties. In general, it has been found that psychrophilic enzymes can help to enhance yields of heat-sensitive products, halophilic enzymes, that are stable in high salt concentrations, serve as models for biocatalysis in low-water media, and thermophilic enzymes are highly resistant to proteases, detergents, and chaotropic agents, which may also afford resistance to the effects of organic solvents [14, 15].  Table 1 lists extremophiles by habitat and some applications of their enzymes.

## 2. SHMT

**Scheme 1:**
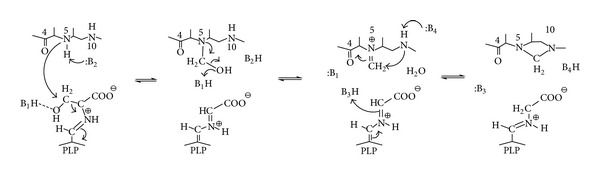
Proposed mechanism for folate-dependent conversion of L-serine to glycine, based on structural information, stereochemical studies, and properties of site mutants. B_1_H is believed to be Glu75 of *e*SHMT [28]. The other catalytic groups are not identified.

 Serine hydroxymethyltransferase (SHMT; EC 2.1.2.1) is a ubiquitous and extensively studied pyridoxal 5′-phosphate-(PLP dependent-) enzyme that catalyzes the reversible transfer of Cβ of L-serine to tetrahydropteroylglutamate (H_4_PteGlu), with formation of glycine and 5,10-methylene-H_4_PteGlu. This reaction is a primary source of the one-carbon units required for the synthesis of thymidylate, purines, and methionine. Moreover, SHMT shows an exceptionally broad substrate and reaction specificity *in vitro*. In fact, with the appropriate substrate analogues, SHMT catalyzes H_4_PteGlu-independent transamination, racemisation, decarboxylation, condensation, and retroaldol cleavage reactions [16, 17]. The rate of the cleavage of a number of 3-hydroxy-amino acids to glycine and the corresponding aldehyde, in some case, approaches and even exceeds the rate of serine cleavage [18, 19]. The increasing availability of solved crystal structures of the enzyme from various prokaryotic and eukaryotic sources [20–24] contributed to clarify a number of observations previously acquired with classical biochemical studies. SHMT belongs to the fold type I group (or aspartate aminotransferase family), which includes many of the best characterized PLP-dependent enzymes. An evolutionary analysis of the fold type I enzymes revealed that SHMT and l-threonine aldolase may actually belong to a subgroup of closely related proteins [25]; fungal alanine racemase, an extremely close relative of l-threonine aldolase, also appears to be a member of the same subgroup [26]. As for the other members of this group, each enzyme subunit, which associates into dimers in prokaryotes and tetramers in eukaryotes, folds into two domains. The active site is located at the interface of the domains and is delimited by amino acid residues contributed by both subunits of the dimer. Several mechanisms have been proposed for the hydroxymethyl transfer [16, 27]. Although the reported crystal structures have provided a wealth of information regarding the architecture of the enzyme, the active site, and the residues involved in substrate binding and catalysis, several aspects of SHMT catalytic mechanism remain uncertain [28]. The currently accepted mechanism for the hydroxymethyltransferase reaction consists of a modified folate-dependent retroaldol cleavage via direct nucleophilic attack of N5 of H_4_PteGlu to Cβ of L-serine, which results in the elimination of the quinonoid intermediate [28, 29] (Scheme 1).

The type of reaction catalyzed by SHMT with different substrate analogues is apparently determined by the structure of the amino acid substrate. With L-serine or glycine, SHMT catalyzes none of the alternative reactions. The currently accepted model attributes this reaction specificity to the existence of an “open” and a “closed” active site conformation, as observed in other members of this family [30]. The physiological substrates trigger the closed conformation, whereas alternative substrates react, while the enzyme remains in the open conformation, which permits alternative reaction paths [31]. The folding mechanism of *Escherichia coli* SHMT has been also investigated and understood in detail [32–34]. It may be divided into two phases and terminates with PLP binding. In the first step, the large and small domains rapidly assume their native state, forming a folding intermediate that is not able to bind PLP. In the second, slower phase, the enzyme folds into the native structure, acquiring the capability to bind the cofactor. Although the crystallographic data have provided a framework for a better understanding of folding studies [35], the key events required for the transition from the first to the second phase remain unclear. Most work on SHMT has focused on enzymes from mesophilic bacteria and eukaryotic organisms. Insights into a better understanding of the structural and functional properties of SHMT could be derived from the studies of the extremophilic enzymes, due to their amazing catalytic characteristics.

SHMT is one of the very few PLP-dependent enzymes that can be found in all living organisms [36], and as it plays a central role in cellular metabolism, it has been repeatedly hailed as a potential target for the development of anticancer and antimicrobial agents [37–39].

## 3. Thermophilic SHMTs

**Figure 1:**
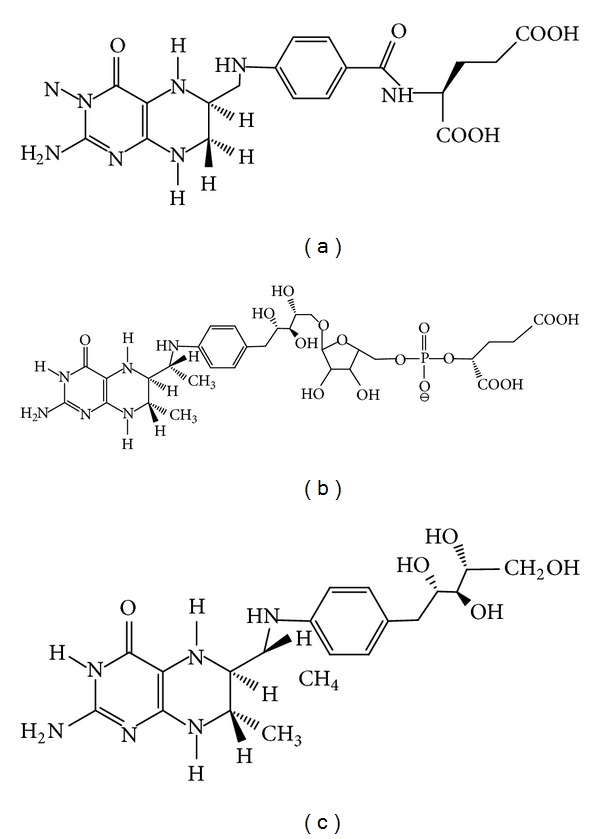
Structures of tetrahydropteroylglutamate (a), tetrahydromethanopterin (b), and sulfopterin (c).

 The thermophilic SHMTs, so far investigated, are present in organisms which belong to the two different kingdoms of life: Archaea and Eubacteria. In Eukarya and Eubacteria, H_4_PteGlu functions as a carrier of C1 units in several oxidation states, which are used in the biosynthesis of important cellular components, such as purines and thymidylate, and in the regeneration of methionine from homocysteine. The reaction catalyzed by SHMT represents in these organisms one of the major loading routes of C1 units onto the folate carrier [27]. In methanogens and several other Archaea, C1 fragments from formyl to methyl oxidation levels are carried by tetrahydromethanopterin (H_4_MPT), a pterin-containing compound involved in methanogenesis [40]. Although H_4_PteGlu and H_4_MPT are structurally similar (Figure 1) and are employed in analogous reactions, most of the H_4_PteGlu-specific and H_4_MPT-specific enzymes are phylogenetically not related. H_4_MPT does not appear to be suited to most of the biosynthetic functions of H_4_PteGlu. Moreover, the biosynthetic pathways of the two carriers have few, if any, homologies, suggesting the possibility of separate evolutionary origins. In the metabolism of folates, SHMT represents a unique link between Archaea and the rest of living beings, in the sense that while all SHMTs clearly share a common evolutionary origin [41], other enzymes which use H_4_MPT as cofactor do not show any significant similarity to their eukaryotic and eubacterial counterparts [40]. Although a gene encoding SHMT is present in all archaeal genomes so far sequenced, little information is available on the catalytic properties, and metabolic role of the enzyme in these organisms. Modified folates are not commercially available and this has clearly hindered a satisfactory characterization of archaeal SHMTs. Moreover, the purification of the enzymes from Archaea which thrive in extreme environments is complicated by the difficulty of growing these organisms in a laboratory.

**Figure 2:**
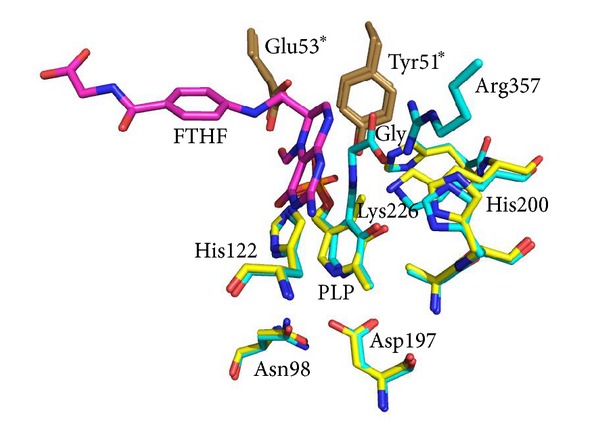
Superimposition of SHMT internal and external aldimine structures. Active site structures of *B. stearothermophilus* SHMT (internal aldimine form: PDB ID 1kkj) and *B. stearothermophilus* SHMT in complex with glycine and 5-formyl-H_4_PteGlu (PDB ID 1kl2) are displayed as yellow and cyan sticks, respectively. The 5-formyl-H_4_PteGlu molecule (FTHF) is displayed as magenta sticks. Side chains from the other subunits are rendered as brown sticks, and the corresponding labels are marked with asterisks. The picture was generated using PyMOL.

Regarding enzymes derived from archaeal organisms, two reports of purified SHMT activity, from *Methanobacterium thermoautotrophicum*, renamed *Methanothermobacter marburgensis *[42], and from *Sulfolobus solfataricus *[43], with limited structural and functional characterization, have been made. In the first report, the enzyme was proposed to function *in vivo *in the direction of L-serine biosynthesis. Both works provided evidence that SHMT was selective towards the modified folates used by the source organisms: H_4_MPT, for *M. marburgensis *and sulfopterin for *S. solfataricus *[40, 44]. More recently, SHMT from the hyperthermophilic methanogen *Methanococcus jannaschii* has been expressed in *E. coli*, purified, and characterized [45]. The results reported in that work suggested that the active site structure and the mechanism of *M. jannaschii* SHMT exhibit no significant differences with respect to their bacterial and eukaryotic counterparts, although the enzyme is characterized by the ability to bind and use the modified folate H_4_MPT as substrate and by an elevated thermal stability. For a better understanding of the functional characteristics of archaeal SHMTs, maybe it would be useful to have the same more structural information, such as the three-dimensional structures of the proteins. Concerning the eubacterial thermophilic SHMTs, two three-dimensional structures are available in Protein Data Bank: one from *Thermus thermophilus *(PDB ID: 2DKJ) and the other from *Bacillus stearothermophilus* (PDB ID: 1KKJ). So far, the best characterized thermophilic SHMT is the enzyme purified from *Bacillus stearothermophilus *(*bst*SHMT), a Gram-positive bacterium, which is able to grow within a temperature range of 30–75°C [46]. The crystal structures of this enzyme have been determined in its internal aldimine form, in binary complex with serine and glycine (external aldimine form), and in ternary complex with glycine and 5-formyltetrahydrofolate (FTHF) [21]. The different structures presented by the authors and the comparison with the other SHMT structures from different sources provide interesting structural information in the reaction mechanism of SHMT. The *bst*SHMT-L-serine complex does not show any significant conformational change compared with the enzyme without bound substrate, contrary to that expected for a conversion from an “open” to “closed” form of the enzyme. However, the ternary complex with FTHF and glycine shows the reported conformational changes. In Figure 2, where active site regions in the internal and external aldimine structures (ternary complex) of *bst*SHMT are depicted, it is possible to see the rotation of PLP ring and the conformational changes of the same active site residues. These small but significant conformational changes are similar to that observed in the structures of the murine cytoplasmic SHMT and *E. coli* SHMT [22, 23].

In contrast to the *E. coli *enzyme, this complex shows asymmetric binding of the FTHF to the two monomers within the dimer in a way similar to the murine SHMT. A detailed analysis of *bst*SHMT structures and a comparison with previously reported structures allow an accurate determination of conformational changes in protein structure, orientation of the PLP ring, and key amino acid residues during different stages of catalysis. An analysis of these results provides structural evidence for a direct transfer mechanism for the SHMT catalyzed reaction (Scheme 1). Further studies on kinetic and structural properties of many *bst*SHMT active-site mutants confirmed these results [47–49]. 

Moreover, an extensive characterization of the structural and functional changes of the *bst*SHMT during unfolding has been carried out [50, 51]. The unfolding properties of the thermophilic enzyme were compared with that of the mesophilic homologues, *Bacillus subtilis *SHMT (*bs*SHMT), with which it shares 77% amino acid sequence identity and with that reported for *E. coli* aspartate aminotransferase (*e*AAT), a mesophilic protein which belongs to the same structural family even sharing a low level of amino acid sequence identity (about 14%) [52]. The results suggest that the *bst*SHMT follows an unfolding mechanism very different from that followed by the *bs*SHMT, despite the high degree of sequence amino acid identity of the two proteins. Instead, the unfolding mechanism of *bs*SHMT is similar to the one followed by the mesophilic aspartate aminotransferase. In fact, *bs*SHMT and *e*AAT undergo a noncooperative unfolding with stabilization of intermediates during the unfolding process, whereas the *bst*SHMT undergoes a highly cooperative unfolding with dissociation of the two monomers and unfolding of native dimer occurring in a single step. Interestingly, preliminary unfolding experiments carried out in our laboratory using the *M. jannaschii* SHMT seem to indicate the same unfolding pathway as the *bst*SHMT (unpublished results).

It would be interesting to compare the kinetic parameters for the folate-dependent and folate-independent reactions of SHMTs from organisms adapted to different temperature. As shown in Table 2, the thermophilic enzyme does not show any enzymatic activity which is significantly different from that shown by the mesophilic enzyme. On the contrary, the kinetic parameters shown by the psychrophilic SHMT, especially that for the folate-independent reactions, suggest that the cold-adapted enzyme is a more suitable catalyst with respect to the mesophilic one (see next paragraph).

## 4. Psychrophilic SHMTs 

 Whereas many theoretical and experimental studies have been devoted to clarify the molecular basis of the adaption of thermophilic enzymes to high temperatures, comparing single thermophilic proteins with their mesophilic counterparts and systematically examining different properties for families of proteins [10, 55, 56], molecular mechanisms of cold adaptation are still relatively unknown. Because of their higher catalytic efficiency at low temperatures, enzymes extracted from psychrophilic organisms have significant potential for biotechnological applications, in particular in industrial processes as energy savers and in detergent industry as additives [57, 58]. The structural adaptation of SHMT synthesized by microorganisms adapted to low temperatures was first investigated using an *in silico* comparative approach, with the aim to detect significant variations of physicochemical properties in SHMTs [59]. Subsequently, the enzyme from psychrophilic microorganism *Psychromonas ingrahamii* was expressed in *Escherichia coli*, purified, and characterized with respect to its spectroscopic, catalytic, and thermodynamic properties [54]. The properties of the psychrophilic enzyme have been contrasted with the homologous counterparts from *E. coli*, which has been extensively, structurally, and functionally characterized [53]. As shown in Table 2, *P. ingrahamii* SHMT (*pi*SHMT) displays higher turnover numbers with respect to *E. coli* SHMT (*e*SHMT), in particular for the side reactions where many substrates, typically *β*-hydroxy-**α**-amino acids, represent important compounds in pharmaceuticals, agrochemicals, and food additives [60]. Most of the comparative studies have been focused on thermal stability. Heat inactivation experiments indicated that *pi*SHMT activity is heat labile, and the apparent melting temperature of the protein is 62°C, which is lower than that of the *e*SHMT [61] (Table 3).

**Figure 3:**
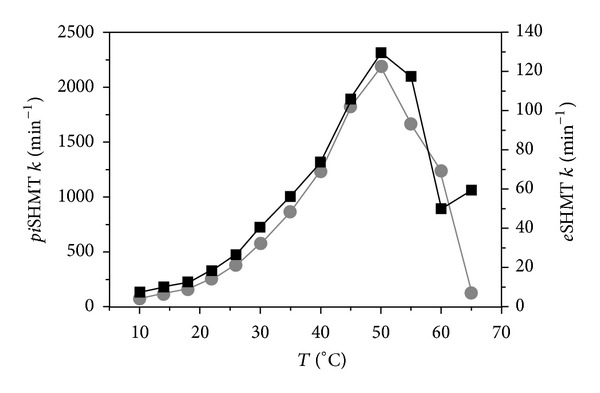
Temperature dependence of enzyme activity of *pi*-(gray dots and left axis scale) and *e*-(black squares and right axis scale) SHMTs. Activities were measured for the retroaldol cleavage of L-*threo*-phenylserine. The picture is adapted from [54].

Interestingly, the difference of the apparent Tm values between the apoform and the holoform of the psychrophilic enzyme is about 20 degrees (Table 3). This observation suggests that the intrinsic instability of the active site is partly compensated by the interaction with the cofactor. The instability and the consequent flexibility of the active site may be functionally relevant also for the conformational transitions it must undergo during the low temperature transfer of the PLP to its binding site within the apoenzyme [34, 62]. Noteworthy, the optimal temperature of enzyme activity of *pi*SHMT is 50°C, which is the same value shown by the *e*SHMT, although the *pi*SHMT activity is at least tenfold higher than *e*SHMT activity (Figure 3).

The relatively high activity characterizing psychrophilic enzymes is the main adaptive parameter to low temperatures and seems to be achieved by the destabilization of the active site or of the entire protein structure, allowing the catalytic center to be more flexible at low temperatures. In this way, the enzyme should be able to reach the transition complex with lower requirement of energy, generally not abundant in a low temperature environment [63]. Recently, articles aimed at finding common structural determinants for cold adaptation have been published (e.g., [11, 13]). Compared with their mesophilic and thermophilic counterparts, cold-adapted enzymes have been reported to share common features such as reduction of the number of Arg, Pro, and Glu and increase in the number of Asn, Gln, Ser, and Met; low Ala/Leu ratio, and lower fraction of larger aliphatic residues expressed by the (Ile+Leu)/(Ile+Leu+Val) ratio; lowered Arg/(Arg+Lys) ratio; reduction in the hydrophobicity of the enzyme; increase of negative charge which facilitates interaction with the solvent; more polar and less hydrophobic residues; fewer hydrogen bond, aromatic interactions, and ion pairs; additional surface loops with more polar residues and lower Pro content. However, no structural feature is present in all cold-adapted enzymes, and no structural feature always correlates with cold adaptation [64]. *pi*SHMT could represent an attractive and interesting enzyme to highlight the structural characteristics coupled to the adaptation to low temperatures, since its structure is very conserved during evolution. Moreover, for its particular catalytic properties, this enzyme is very promising in biotechnological applications (see next paragraph). 

## 5. Extremophilic SHMTs as a Valuable Tool for Biotechnological Applications

**Scheme 2:**
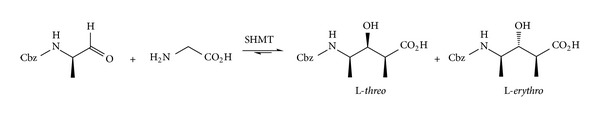
SHMT-catalyzed aldol addition of glycine to (R)-N-Cbz-alaninal.

 With the steady growth of the importance of enantiomerically pure or enriched compounds in pharmaceuticals, agrochemicals, and food additives, the so-called “chiral market” has become an expanding area of the fine chemicals industry [65]. In particular, β-hydroxy-*α*-amino acids are an important class of natural products that have recently received much attention due to their biological activity on their own and as constituents of many naturally occurring complex compounds, such as antibiotics and immunosuppressants. For example, L-*threo*-β-(3,4-dihydroxyphenyl) serine is a special remedy in the Parkinson's disease treatment as an agent for norepinephrine precursor therapy [66], L-*threo*-*α*-(4-methylthiophenyl) serine is an intermediate for the production of antibiotics, such as florfenicol and thiamphenicol [67, 68], 4-hydroxy-L-threonine is a precursor of rhizobitoxine, and 3,4,5-trihydroxy-L-aminopentanoic acid is a key component of polyoxins [69]. Furthermore, the β-hydroxy-*α*-amino acids, as polyfunctional compounds, might be useful for building blocks for peptidomimetics and other nonproteinogenic peptide-like molecules of biological interest. β-hydroxy-*α*-amino acids can be obtained through the asymmetric chemical synthesis. Hayashi and Belokon carried out a series of fundamental and creative research in this field; for example, they have investigated asymmetric aldol reactions of isocyanoacetic derivatives with fluoroaryl aldehydes, benzaldehydes, and aryl ketones catalyzed by gold (I) or silver(I)/triethylamine [70, 71]. In addition to asymmetric synthesis, β-hydroxy-*α*-amino acids are mainly produced through chemical synthesis processes, followed by chiral resolution [72]. These processes however have some drawbacks, such as the following: chiral resolution is time-consuming and inefficient, and the overuse of organic solvents results in environmental problems. Accordingly, the development of an efficient and clean enzymatic resolution process is desirable. As discussed previously, SHMT catalyzes the cleavage of several C3-OH amino acids varying in substituent and stereochemistry at C3, with most research focusing on threonine and β-phenylserine [18, 19]. None of these reactions requires H_4_PteGlu as a cosubstrate, and the rates approach or exceed the rate of H_4_PteGlu-dependent serine cleavage. Therefore, SHMT represents a good tool for biotechnological applications. Extensive studies have been carried out on the biotransformation activity of serine hydroxymethyltransferase from different species. The SHMTs extracted from *H. methylovorum* and from *E. coli* were found to have a wide substrate specificity. Regarding the degradation of the β-hydroxy-*α*-amino acids, β-*threo*-phenylserine, L-serine, *allo-*threonine, *threo*-3,4-dihydroxy-phenylserine, and L-threonine were good substrates [53, 73]. SHMT also showed potential as a biocatalyst for the stereoselective synthesis of β-hydroxy-*α*-amino acids. In [74] the authors described the aldol addition of glycine to nonnatural aldehydes, such as benzyloxyacetaldehyde and (R)-N-Cbz-alaninal (Cbz = benzyloxycarbonyl) to corresponding β-hydroxy-*α*-aminoacid diastereoisomers catalyzed by the recombinant SHMT derived from the *Streptococcus thermophilus *YKA-184 strain. The reaction described in that work shows a moderate stereospecificity concerning the β-carbon, with diastereomeric ratio of 80 : 20 between the L-*threo * isomer versus L-*erythro* isomer (Scheme 2).

**Scheme 3:**
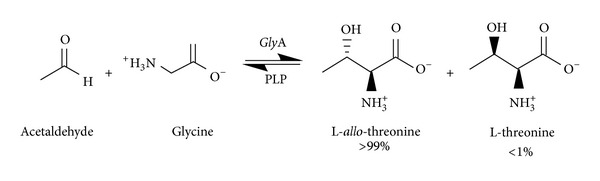
*Gly*A-catalyzed aldol reaction of glycine and acetaldehyde in the presence of the cofactor PLP.

In [75], the authors investigated the effect of reaction variables, such as temperature, reaction media, and glycine concentration on this aldol addition reaction and the diastereomeric ratio, with the aim to obtain a better enzymatic reaction performance and to further exploit the synthetic utility of this enzyme. In particular, it has been shown that temperature is an important parameter. In fact, at low temperatures, the retroaldol activity is strongly inhibited, whereas a high synthetic capacity is maintained. Thus, it might be synthetically useful to work at low temperatures. Moreover, in [76], it has been reported the diastereospecific formation of L-*allo*-threonine catalyzed by an *E. coli* strain harbouring serine hydroxymethyltransferase gene (*gly*A gene) (Scheme 3).

These results show that SHMT could be a promising biocatalyst for the stereoselective synthesis of β-hydroxy-*α*-amino acids. Such industrial processes would benefit from the employ of SHMTs that function at extreme temperature. Generally, thermophilic enzymes offer economic advantages such as increased rates of reactions, a higher substrate solubility, and/or longer enzyme half-lives at the conditions of industrial processes. On the other hand, psychrophilic enzymes provide other important benefits through energy savings: they exhibit increased reaction yields in cold environments, a high level of stereospecificity, an increased thermal lability for rapid and easy enzyme inactivation when required and minimization of undesirable chemical reactions that can occur at higher temperatures. In particular, the structural and functional properties of the cold-adapted SHMT from *Psychromonas ingrahamii* described in [54] are especially promising for biotechnological applications. This enzyme, in fact, is a more efficient biocatalyst compared to the other SHMTs, especially for the side reactions involving β-hydroxy-*α*-amino acids as substrates. However, further investigations are required for a better understanding of the catalytic properties and, in particular, the stereospecificity of *pi*SHMT. Stereospecificity in cold-adapted enzymes has been poorly investigated. It has been reported [77] that psychrophilic enzymes seem to be more stereoselective with respect to meso/thermophilic homologs, although it is not completely clear how the high flexibility of their molecules can be related to this peculiarity.

## 6. Conclusions

 The synthesis of polymer precursors, pharmaceuticals, specialty chemicals, and agrochemicals is often hampered by expensive processes that suffer from low selectivity and undesired byproducts. Mesophilic enzymes are often not well suited for the harsh reaction conditions required in industrial processes because of the lack of enzyme stability. The recent advances in the study of the stable enzymes from extremophiles have resulted in their increased use for applications in the organic synthesis. Our understanding of the biochemical and structural properties of the extremophilic SHMTs, coupled to enzyme modification by rational protein engineering or directed evolution, could lead to improved catalytic and physical properties and the development of novel catalytic functions.

## Figures and Tables

**Table 1:** Industrial applications of enzymes isolated from extremophiles.

Extremophiles	Habitat	Enzymes	Representative applications
Thermophile	High temperature	Amylases Xylanases Proteases DNA polymerases	Production of glucose, fructose for sweeteners Paper bleaching Used in baking, brewing, and as detergent additive Genetic engineering

Psychrophile	Low temperature	Proteases Dehydrogenases Amylases	Cheese maturation Biosensors Polymer degradation in detergents

Acidophile	Low pH	Sulfur oxidation	Desulfurization of coal

Alkalophile	High pH	Cellulases	Polymer degradation in detergent

**Table 2:** Comparison of the kinetic parameters of the reactions catalyzed by different SHMTs.

Substrate	*E. coli* SHMT^a^		SHMT^a^ * P. ingrahamii *		*B. stearothermophilus* SHMT^b^	
	*K*_*m*_ (mM)	*k*_cat_ (min^−1^)	*K*_*m*_ (mM)	*k*_cat_ (min^−1^)	*K*_*m*_ (mM)	*k*_cat_ (s^−1^)
L-Threonine	43^d^	4.3^d^	20.2^f^	6.6^f^	ND^c^	ND^c^
L-*Threo-*phenylserine	19^d^	167^d^	17.2^f^	852^f^	ND^c^	ND^c^
L-*Allo-*threonine	1.5^e ^	30^e ^	1.6^f^	107^f^	0.9^g^	0.47^g^
L-Serine	0.3^e ^	640^e^	0.4^f^	555^f^	0.9^g^	3.9^g^

^a^Reactions were carried out at 30°C

^b^Reactions were carried out at 37°C

^c^Not determined

^d^From [25]

^e^From [53]

^f^From [54]

^g^From [48].

**Table 3:** Apparent melting temperatures (Tm) of different SHMTs.

	Tm of apoenzymes (°C)	Tm of holoenzymes (°C)	Tm in presence of L-Serine (°C)	Reference
*E. coli* SHMT	58.8	69.5	73.0	[61]
*P. ingrahamii* SHMT	42.0	62.0	ND^a^	[54]
*B. stearothermophilus* SHMT	ND^a^	65.0	74.0	[48]

^a^Not determined.

## Abbreviations

H_4_PteGlu: Tetrahydrofolate or tetrahydropteroylglutamate
H_4_MPT: Tetrahydromethanopterin
FTHF: 5-Formyl tetrahydrofolate
PLP: Pyridoxal 5′-phosphate
*e*SHMT: *Escherichia coli* serine hydroxymethyltransferase
*pi*SHMT: *Psychromonas ingrahamii* serine hydroxymethyltransferase
*bst*SHMT: *Bacillus stearothermophilus* SHMT
*bs*SHMT: *Bacillus subtilis* SHMT
*e*AAT: *Escherichia coli* aspartate aminotransferase.
